# Development and internal validation of multimodal machine learning models for predicting eligibility for mechanical thrombectomy in suspected stroke patients using routinely collected clinical and imaging data

**DOI:** 10.1371/journal.pone.0334242

**Published:** 2025-10-10

**Authors:** Arjun Agarwal, Nirman Bharti, Tamaghna Ghosh, Satish Golla, Navpreet K. Bains, Rashi Chamadia, Dennis Robert, Preetham Putha, Adnan I. Qureshi

**Affiliations:** 1 Qure.ai Technologies Private Limited, Mumbai, India; 2 Qure.ai Technologies Private Limited, Bangalore, India; 3 Zeenat Qureshi Stroke Institute, St. Cloud, Minnesota, United States of America; 4 University of Missouri, Columbia, United States of America; Fondazione Policlinico Universitario Agostino Gemelli IRCCS, ITALY

## Abstract

**Background:**

Mechanical thrombectomy (MT) eligibility for acute ischemic stroke (AIS) patients depends upon clinical and advanced imaging assessments like CT perfusion (CTP). Assessment complexities and limited access to advanced imaging investigations are known challenges. We developed machine-learning models using routinely collected clinical and imaging data to predict MT eligibility.

**Methods:**

Age, National-Institutes-of-Health-Stroke-Scale-Score (NIHSS), last-known-well-time (LKWT), noncontrast-CT (NCCT) scan and CT-angiography (CTA) report from consecutive cohort of 260 AIS-suspected patients treated at a stroke centre during Apr’20 to Dec’23 were retrospectively collected. 160 underwent MT for anterior-circulation large vessel occlusion (LVO_a_); rest were MT ineligible. MT eligibility was determined based on clinical and imaging investigations including CTP during routine-care. The dataset was split into train:test sets (50:50 split). A commercially available artificial-intelligence algorithm calculated infarct volume and ASPECT score (ASPECTS_q_) from the NCCTs. We developed two supervised models using Gradient-Boosting-Machines. MODEL_1_ utilized age, NIHSS, LKWT, ASPECTS_q_ and infarct volume as inputs; MODEL_2_ additionally included the presence/absence of LVO_a_ as input. The target/response variable used for our supervised learning methods was whether the patients were MT eligible or not as determined during routine-care. Performance of the models were investigated using the test set.

**Results:**

Among 130 patients (mean age ± standard-deviation: 67.4 ± 14.2 years; 61 males) in test set, 80 (61.5%) were MT eligible; rest were ineligible. The area-under-the-receiver-operating-characteristics-curve, sensitivity and specificity of MODEL_1_ were 0.76 (95% CI: 0.67–0.85), 85% (75.6–91.2) and 60% (46.2–72.4), respectively. They were 0.92 (0.88–0.96), 82.5% (72.7–89.3) and 82% (69.2–90.2), respectively, for MODEL_2_.

**Conclusions:**

The models showed promising results, demonstrating that NCCT, potentially with CTA, could be sufficient for MT eligibility determination. Such models can enable faster referrals of patients to higher centers.

## Introduction

Mechanical thrombectomy (MT) is established as a treatment of choice in acute ischemic stroke (AIS) patients with anterior circulation large vessel occlusion (LVO_a_) [1]. Whilst initially MT was recommended for patients with LVO_a_ within 6 hours of last known well time (LKWT) and with Alberta Stroke Program Early Computed Tomography Score (ASPECTS) ≥ 6, randomized controlled trials have demonstrated benefit of MT in eligible patients within 6–24 hours of LKWT, with consequent recommendations in the guidelines [1–3]. Eligibility for MT in patients presenting during the extended window (within 6–24 hours of LKWT) is extrapolated from randomized controlled trials [2,3] utilizing imaging modalities such as Computed Tomography Perfusion (CTP) or Diffusion-weighted Magnetic Resonance Imaging (DW-MRI), while for those presenting within 6 hours of LKWT, eligibility for MT can be determined based on non-contrast CT (NCCT) brain and CT angiogram (CTA) or MRI/MR angiogram alone [1]. Recently, another randomized controlled trial has shown superior effectiveness of MT in large core infarct (ASPECTS score ≤ 5) LVO_a_ patients presenting within 6.5 hours of LKWT compared to medical care alone [4].

While CTP and DW-MRI imaging are largely used in advanced stroke care centers for MT eligibility in suspected AIS patients, these advanced imaging modalities have limited availability in under-resourced settings, and require qualified personnel to interpret imaging studies and post-processing software to generate CTP parameter maps which are further complicated by variability in parameter maps generated by different commercially available software packages [5–7]. Though the utilization of CTA and CTP for suspected stroke patients are on the rise, data suggests that they are still underutilized [8]. As a consequence, many otherwise eligible patients may not undergo MT or experience delay in undergoing MT which can adversely affect their outcomes. It is thus pertinent to investigate if eligibility for MT in AIS suspect patients can be accurately predicted by using data without using advanced imaging investigations. Such models could potentially assist concerned healthcare professionals in clinical decision making, especially resource constrained settings. While numerous pre-hospital models exist in literature, most are limited by being unimodal, focusing on predicting LVO_a_ likelihood rather than MT eligibility [9]. Additionally, these models often have suboptimal inter-rater reliability and require complex assessments that are challenging for pre-hospital personnel to implement [9].

In this study, we report development and internal validation of machine learning models using multimodal data from routinely collected patient data and imaging modalities such as NCCT and/or CTA that would predict MT eligibility.

## Materials and methods

### Data collection

For the development and validation of the multimodal prediction model (s), we retrospectively collected anonymized data from 260 consecutive stroke-suspect patients aged 18 years and older admitted to an advanced stroke care center in the United States of America (Zeenat Qureshi Stroke Institute, St. Cloud, Minnesota, USA) between April 2020 and December 2023. All the 260 patients underwent stroke evaluation with National Institutes of Health Stroke Scale (NIHSS) [10], NCCT, CTA and CTP and had a LKWT of maximum 24 hours. All imaging investigations were performed within 24 hours of symptoms onset. The radiologist interpretations of CTA and CTP parameter maps from a CTP post-processing software package were in the collected data, but not CTA and CTP scans. NCCT scans were available. 160 (61.5%) of the patients underwent MT with eligibility determined by clinical, imaging and CTP perfusion parameters. Of the remaining 100 (38.5%) patients who did not undergo MT, 30 were found ineligible for MT despite presence of LVO_a_ and the remaining 70 did not have a LVO_a_. Patients with missing data on age, NIHSS and imaging (NCCT, CTA and CTP) data were excluded.

### Model development

#### Training data.

**Fig 1:**
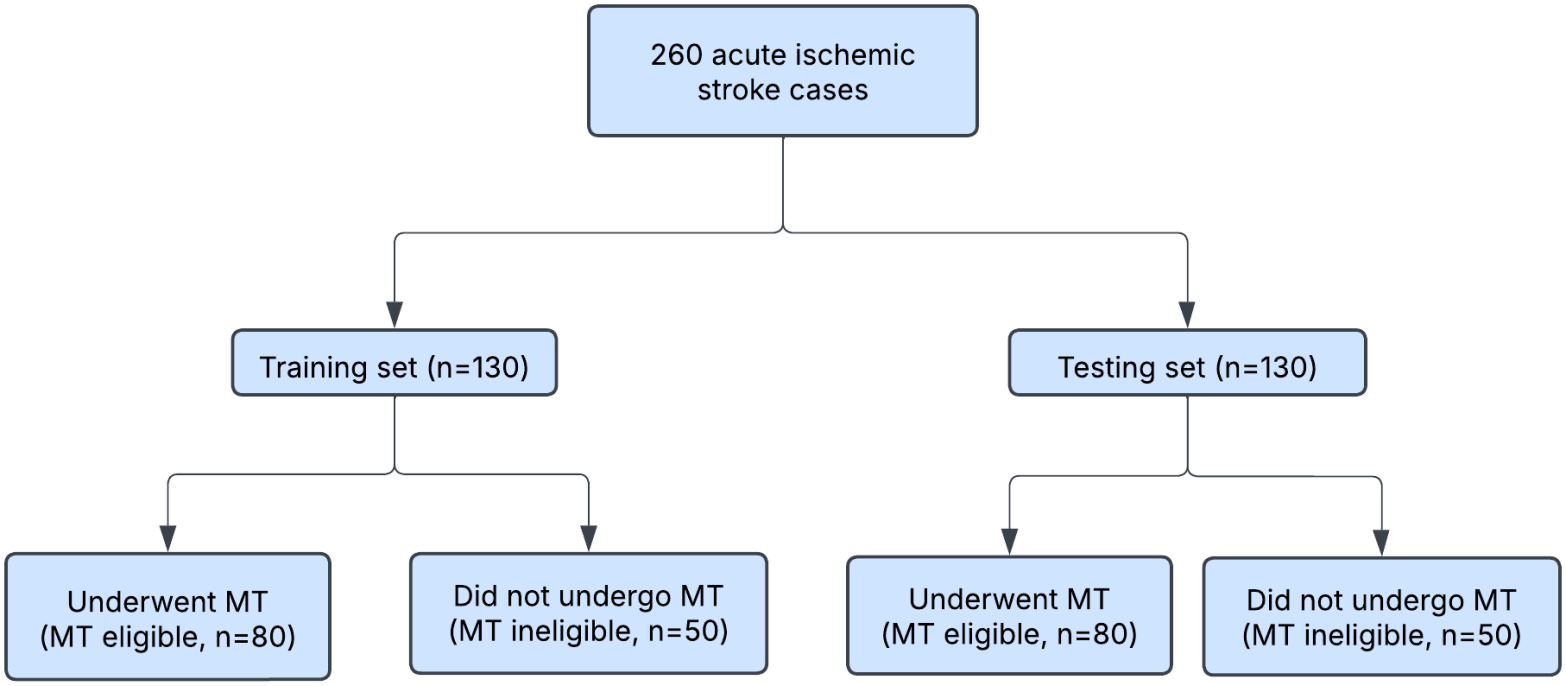
Data Flow Diagram.

We used data from 130 patients (80 who underwent MT [MT eligible]) and 50 who did not) for training the multimodal prediction model and reserved the remaining 130 (50:50 train:test split) as testing dataset (Fig 1). The target/response variable used for our supervised learning was based on whether the patients underwent MT (MT eligible, n = 80) or not (MT ineligible, n = 50).

#### Predictor variables.

**Fig 2:**
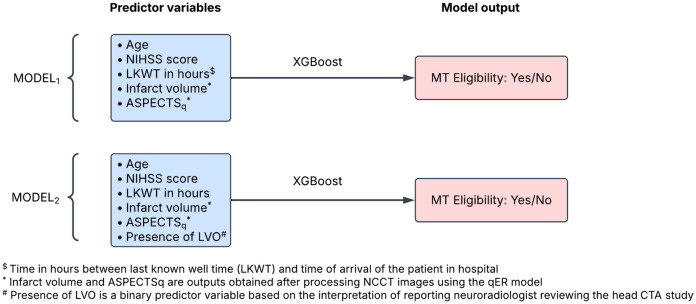
Illustration of predictor variables and model output of the two XGBoost (eXtreme Gradient Boosting) multimodal prediction models. ASPECTS: Alberta Stroke Program Early Computed Tomography Score.

We developed two supervised binary classification prediction models, named MODEL_1_ and MODEL_2_, using the eXtreme Gradient Boosting (XGBoost) machine learning algorithm [11]. Predictor variables of MODEL_1_ included five predictor variables – 1) age, 2) LKWT, 3) NIHSS score, 4) infarct volume in millilitre (ml) predicted by an existing deep learning based segmentation model (qER, Qure.ai) from NCCT scan [12–15] and 5) ASPECT score (ASPECTS_q_) predicted by qER from NCCT scan (Fig 2). MODEL_2_ included an additional binary predictor variable for the presence or absence of LVO_a_ based on the CTA interpretation of the original reporting neuroradiologist. Details of the predictor variables based on NCCT scans (infarct volume and ASPECTS_q_) are detailed below.

##### Infarct volume:

We utilized an existing acute infarct segmentation model (qER; Manufacturer: Qure.ai) to determine infarct volume. This model is a semantic segmentation-based deep learning framework that employs a transformer-based encoder architecture, paired with a robust segmentation head. The encoder is pretrained on 125,000 head NCCT scans to classify the presence of infarcts, distinguishing between acute and chronic infarcts. The segmentation head leverages the encoder’s feature representations to generate three target masks: all infarcts, acute infarcts, and chronic infarcts. The final output is a refined segmentation map that accurately delineates infarcted regions. For our analysis, we focused on the volume of the acute infarct mask, as our focus was on acute stroke cases.

##### ASPECTS_q_:

**Fig 3:**
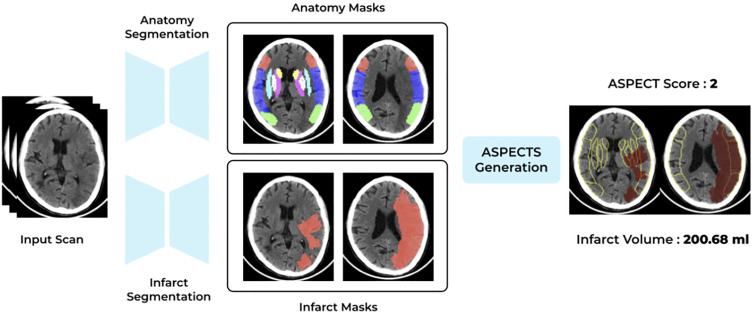
qER ASPECT score model.

qER’s ASPECT score model combines two components: the infarct segmentation model and the ASPECT anatomy model. The ASPECT anatomy model predicts a mask of the 10 regions associated with ASPECT scoring, while the infarct segmentation model (described in section above) predicts the acute infarct mask (Fig 3). When integrated, these models provide an efficient means to quickly obtain the ASPECT score (a value between 0 and 10) for any NCCT scan. For reporting purpose, we call this ASPECT score outputted from qER based on processing the NCCT scan as ASPECTS_q_. The ASPECT anatomy model was trained on an extensive dataset of 50,000 head NCCT scans.

### XGBoost models

Our multimodal prediction models (MODEL_1_ and MODEL_2_) were built using XGBoost algorithm. XGBoost is a widely used powerful ensemble machine learning algorithm known for its high performance in classification tasks, particularly in scenarios involving complex and heterogeneous data, such as in medical decision-making [11,16]. By utilizing an ensemble of decision trees, XGBoost can capture non-linear relationships and interactions between the predictor variables, making it an ideal choice for our model’s classification task. XGBoost is reported to produce accuracies which are comparable to many state of the art machine learning algorithms, including deep neural networks [16].

In our study, the XGBoost algorithm was trained to model the probability for MT eligibility based on the predictor variables in the training data described earlier. Multiple hyperparameters of the XGBoost model, such as learning rate, tree depth, minimum child weight, subsample ratio and feature sampling were optimized through grid search to find the best hyperparameter values. The final model configuration was selected based on optimal MT eligibility area under the receiver operating characteristics curve (AUROC) performance. A fixed random state was used to ensure reproducibility across experiments.

### Model testing and statistical analysis

A sample size of 130 patients (80 MT eligible and 50 MT ineligible) would be enough to estimate an AUROC of 0.75 or more with minimum 9% precision (half-width of 95% CI) [17]. The fine-tuned MODEL_1_ and MODEL_2_ were evaluated using the testing set consisting of data from 130 patients (80 who underwent MT [MT eligible]) and 50 who did not). The reference standard (ground truth) used for evaluation was based on whether the patients were deemed MT eligible or not during their assessment at the stroke care center. AUROC, sensitivity and specificity of the models are reported along with their 95% confidence intervals. To calculate sensitivity and specificity, an optimal threshold maximizing sensitivity and specificity was applied to the probability score for each model in order to classify the instances into MT eligible or not. We also report accuracy (proportion of correctly classified instances) and F1 score (harmonic mean of positive predictive value and sensitivity). We also performed the feature (predictor variable) importance analysis and SHapley Additive exPlanations (SHAP) [18] analysis of predictor variables.

Since MODEL_1_ and MODEL_2_ also used infarct volume and ASPECTS_q_ as predictor variables, which were in turn based on prediction outputs from an existing deep learning model (qER), we performed a comparison of qER’s acute infarct prediction with radiologists as an exploratory analysis. For this analysis, all 260 head NCCT scans (training and testing data combined) were independently reviewed by four radiologists (one neuroradiologist and three general radiologists, 7–15 years range of experience) blinded to any other information to discern the presence/absence of acute infarct. The sensitivity and specificity of the radiologists in detecting acute infarct were descriptively compared with that of qER.

### Ethical considerations

The study proposal was reviewed by Institutional Review Board (IRB) at University of Missouri-Columbia (IRB Project Number: 2095636) and was deemed exempt for review. Informed consent was not required due to the retrospective nature of the study. Only the authorized investigators (NKB and AIQ) from the institution had access to identifiable information during data collection. Any identifiable information was permanently removed immediately after data collection and only de-identified data was used for model development and validation. Data were accessed during February 2023 to March 2024 in accordance with annual IRB exemption requirements.

## Results

### Model training

Baseline characteristics of the 130 patients included in the training data are shown in Table 1. Best performing models after hyperparameter tuning had 10 decision trees for MODEL_1_ and 12 for MODEL_2_ and a maximum depth of 3 for both the models, preventing overfitting while maintaining sufficient complexity. A minimum child weight of 2 to control tree splitting, a subsample ratio of 0.6 to introduce randomness for better generalization, and 60% feature sampling to reduce feature correlation were also fine-tuned through grid search for both models. Learning rate was found to be optimal at 0.15 for MODEL_1_ and for 0.10 for MODEL_2_. The best checkpoint yielded an AUROC of 0.86 (95% CI: 0.80–0.92) for MODEL_1_ and 0.96 (95% CI: 0.93–0.99) for MODEL_2_.

**Table 1:** Baseline characteristics of patients in the training dataset.

Baseline characteristic	Overall (n = 130)	MT eligible (n = 80)	MT ineligible^*^ (n = 50)
**Age in years**			
Mean ± SD	66.4 ± 17.0	68.0 ± 15	63.8 ± 19.6
Median (IQR)	68 (57-77.75)	68.5 (58.5-78)	67 (55-75.75)
**Gender**			
Males, N (%)	74 (56.9%)	45 (56.2%)	29 (58%)
**NIHSS score**			
Median (IQR)	12 (6.25-15.75)	14(10.0-17.0)	6 (2.25-14.00)
**Presence of LVO**_**a**_			
N (%)	95 (73%)	80 (100%)	15 (30%)
**LKWT**			
Less than 6 hours: N (%)	74 (56.9%)	45 (56.2%)	29 (58%)
6–24 hours: N (%)	56 (43.1%)	35 (43.8%)	21 (42%)

SD, standard deviation; IQR, inter-quartile range. N, absolute number; LVO_a_, anterior circulation large vessel occlusion; LKWT, last known well time.

* MT ineligible group included patients with LVO_a_ (n = 15) and with no LVO_a_ (n = 35).

### Multimodal prediction model performance on testing data

The mean age of the patients in the testing data was 67.4 years (standard deviation: 14.2 years) and 61 (46.9%) were males. The baseline characteristics of patients in the testing data are detailed in Table 2.

**Table 2:** Baseline characteristics of patients in the testing dataset.

Baseline characteristic	Overall (n = 130)	MT eligible (n = 80)	MT ineligible^*^ (n = 50)
**Age in years**			
Mean ± SD	67.4 ± 14.2	66 ± 14.6	69.6 ± 13.4
Median (IQR)	67.5 (59.25-77)	67 (56.75-77)	69 (61-77.5)
**Gender**			
Males, N (%)	61 (46.9%)	40 (50%)	21 (42%)
**NIHSS score**			
Median (IQR)	11.5 (5-18)	14 (8-18)	5 (2-15.5)
**Presence of LVO**_**a**_			
N (%)	95 (73.1%)	80 (100%)	15 (30%)
**LKWT**			
< 6 hours: N (%)	89 (68.5%)	56 (70%)	33 (66%)
6–24 hours: N (%)	41 (31.5%)	24 (30%)	17 (34%)

SD, standard deviation; IQR, inter-quartile range. N, absolute number; LVO_a_, anterior circulation large vessel occlusion; LKWT, last known well time.

* MT ineligible group included patients with LVO_a_ (n = 15) and without LVO_a_ (n = 35).

**Fig 4:**
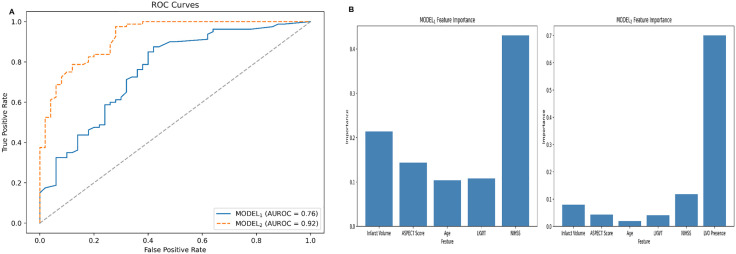
Receiver operating characteristics (ROC) curve (A) and bar plot of feature importance (B) of both models.

MODEL_1_, which incorporates clinical data and results from NCCT scans, achieved an AUROC of 0.76 (95% CI: 0.67–0.85) (Table 3). In contrast, MODEL_2_, which integrates clinical data with results from both NCCT and CTA (presence of LVO_a_), demonstrated an AUROC of 0.92 (95%CI: 0.88–0.96) (Fig 4). The performance of both the models was better in patients with LKWT of less than 6 hours (AUROC: 0.78 [95% CI: 0.67–0.88] for MODEL_1_ and 0.95 [95% CI: 0.90–0.99] for MODEL_2_) compared to that in patients with LKWT of 6–24 hours (AUROC: 0.65 [95% CI: 0.47–0.82] for MODEL_1_ and 0.86 [95% CI: 0.73–0.95] for MODEL_2_). The improvement in AUROC of MODEL_2_ compared to MODEL_1_ is reflected further with a larger improvement in specificity (82% for MODEL_2_ and 60% for MODEL_1_) than improvement in sensitivity (82.5% for MODEL_2_ and 85% for MODEL_1_). The feature importance analysis showed that NIHSS score and infarct volume were the top two predictor variables in MODEL_1_ whereas in MODEL_2_ these were the presence of LVO_a_ and NIHSS (Fig 4). The top predictor variable as per SHAP analysis was NIHSS score for MODEL_1_ (S1 Fig) and presence of LVO_a_ for MODEL_2_ (S2 Fig) which were consistent with the feature importance. However, the ranking of the other predictor variables was not similar in order between feature importance analysis and SHAP analysis. This could be because feature importance analysis considers how much each feature contributes to model predictions overall and SHAP analysis considers how much each feature contributes to prediction of each individual instance.

**Table 3:** Performance results of the multimodal prediction models in predicting MT eligibility in the testing set.

Metric	Overall (n = 130)	LKWT of less than 6 hours (n = 89)	LKWT of 6–24 hours (n = 41)
**MODEL**_**1**_			
AUROC	0.76 (0.67-0.85)	0.78 (0.67-0.88)	0.65 (0.47-0.82)
Sensitivity	85.0% (75.6-91.2)	85.7 (74.3-92.6)	83.3% (64.1-93.3)
Specificity	60.0% (46.2-72.4)	63.6 (46.6-77.8)	52.9% (31.0-73.8)
Accuracy	75.4% (67.3-82.0)	77.5 (67.8-85.0)	70.7% (55.5-82.4)
F1 score	0.810	0.828	0.769
**MODEL**_**2**_			
AUROC	0.92 (0.88-0.96)	0.95 (0.90-0.99)	0.86 (0.73-0.95)
Sensitivity	82.5% (72.7-89.3)	91.1% (80.7-96.1)	62.5% (42.7-78.8)
Specificity	82% (69.2-90.2)	84.8% (69.1-93.3)	76.5% (52.7-90.4)
Accuracy	82.3% (74.8-87.9)	88.8% (80.5-93.8)	68.3% (53.0-80.4)
F1 score	0.852	0.911	0.698

AUROC, area under the receiver operating characteristics curve; LKWT, last known well time.

### Comparison of acute infarct detection in head NCCT scans between qER and radiologists

Of the 260 patients, 190 (73.1%; 160 [84.2%] MT eligible and 30 [15.8%] MT ineligible) had confirmed presence of LVO_a_ and thus were considered to have presence of acute infarct. The rest of the 70 patients had no LVO_a_ and no perfusion deficit and thus were considered as controls (no acute infarct) in this exploratory analysis. Overall, the sensitivity of the radiologists ranged from 5.0% to 21.2% whereas that of qER was found to be 25.0% (S1 Table and S3 Fig). The specificity of the radiologists ranged from 78.6% – 98.6% and that of qER was found to be 85.7%.

## Discussion

In this study, we trained multimodal prediction models using clinical and imaging data from head NCCT and CTA scans to predict MT eligibility in a patient with AIS. Using only clinical data and basic imaging investigation (NCCT), the model achieved a good AUROC of 0.76 (95% CI: 0.67–0.85) in testing set. When neuroradiologist’s interpretation on the presence or absence of LVO_a_ from head CTA was added as a new predictor variable, the model had significant improvement in performance as indicated by an excellent AUROC of 0.92 (95% CI: 0.88–0.96). At optimal threshold, the specificity of MODEL_2_ was much higher than that of MODEL_1_, but the sensitivities of the two models were not that largely different.

The benefit of MT is known to be time dependent. For every 15-minute reduction in door-to-reperfusion time during MT, an estimated 39 out of 1,000 patients show improved outcomes at 3 months [19]. The economic and clinical implications of accurate MT eligibility prediction are substantial. Unnecessary delays in treatment can significantly increase neurological disability, healthcare costs, and long-term patient burden. Delay in transferring patients from primary centres to comprehensive stroke care centres and requirement of CTP may contribute to delay in access to MT [20,21]. Our models could potentially be useful for suspected AIS patients, especially those presenting initially in low-resource settings without advanced imaging infrastructure such as CTP or DW-MRI, enabling faster referral to higher stroke care centers. The predictor variables in our models would already be routinely collected as part of basic assessment of stroke suspect patients. In a real-world setting, these models could act as triaging tools for enabling faster referrals. Since false negative (incorrectly classifying an MT eligible patient as ineligible) results are likely to cause worse outcomes for patients than false positive results, a high sensitivity is preferable for such models even at the expense of specificity. These models can be technically integrated into the hospital information technology infrastructure so that real-time model prediction results can be viewed by concerned healthcare professionals such as emergency care physicians, radiologists, neurologists and/or neurointerventional professionals, either within hospital or remotely.

To our knowledge, this is the first multimodal MT eligibility prediction model of its kind described in the literature. There are several unimodal prediction models available to assess the likelihood for LVO_a_ in pre-hospital setting using basic clinical data alone [9]. However, not all LVO_a_ patients are eligible for MT. Koster et al. conducted a systematic review to assess the effectiveness of several such models in determining the eligibility for MT and observed that the AUROCs of these models ranged from 0.75 to 0.83 [22]. Interestingly, NIHSS score is reported to have an AUROC of 0.81 which is comparatively higher than the AUROC of our MODEL_1_ which also included NIHSS score as one of the predictor variables. NIHSS score was the top predictor variable in MODEL_1_. A single center data and the relatively small size of the training dataset in our study may be the reason for the lower AUROC in our MODEL_1_. However, the addition of a new predictor variable indicating presence/absence of LVO_a_ significantly improved model performance to 0.92 which is comparatively higher than the best performing pre-hospital clinical prediction model (FAST-ED). FAST-ED, however, suffers from suboptimal interrater reliability and uses complex items which are difficult for pre-hospital care personnel to assess [23,24]. Seetge et al. used a logistic regression model using age, NIHSS score at admission, and pre-morbid modified Rankin Scale score as predictor variables to stratify AIS patients into low-, moderate-, and high-risk groups, to guide treatment decisions on thrombolysis, MT, combination therapy (thrombolysis + MT), or standard care, and observed that the model had an AUROC of 0.86 in predicting 90-day outcomes [25]. Alwood et al. used CTP parameter map outputs from two CTP post-processing software packages and reported that there were significant differences in core and penumbra volume estimates between the outputs of software packages [26]. They also found that there were no statistically significant differences between the two software packages when they were used to determine MT eligibility based on the DEFUSE-3 [3] eligibility criteria. Our models incorporate simple clinical data which are routinely collected in hospitals and outputs from imaging data which can be fed into the model in real-time when scans are acquired and/or reported. We acknowledge that MODEL_2_, however, requires input from a head CTA study which may not be available in many low-resource settings and that comparison of pre-hospital clinical prediction models with in-hospital prediction models may not be very ideal.

In our exploratory analysis of comparing acute infarct detection performance of radiologists with our existing deep learning model (qER) in NCCT scans, we observed better acute infarct detection rates for qER. The sensitivity of both radiologists and qER were comparatively higher in NCCT scans from patients who were ineligible for MT despite LVO_a_. These patients were ineligible for MT due to established hypodensity on NCCT suggestive of established infarct and thus might have been easier to detect. The sensitivity in detecting AIS from NCCT scans is generally considered to be on the lower side with one study reporting it to be about 26% [27] and in general considered to be around 40% [28]. It is noteworthy that the sensitivity of qER in detecting acute infarct was 40% in MT eligible patients and 60% in MT ineligible (but with presence of LVO_a_) patients.

Major limitations of our study include using a single center data, relatively smaller sample size in the training data and use of testing data from the same source as that of the training data. In future work, we intend to retrain models using data from multiple sources and use independent external data sources for performing external validation to improve the generalizability of the models. We only experimented with XGBoost machine learning algorithm, and it can be argued that other machine learning algorithms such as, but not limited to, random forests and logistic regression, could also have been experimented with. We chose XGBoost in this study because it is well known to be amongst the best performing machine learning algorithms. For MODEL_2_, we ideally could have utilized another existing head CTA LVO_a_ prediction algorithm using CTA scans as input to generate the predictor variable for the presence of LVO_a_, but we did not have access to CTA imaging data and thus had to rely on reporting neuroradiologist’s interpretation as the predictor variable.

## Conclusions

Our multimodal prediction models show potential in predicting the eligibility for MT using routinely collected clinical and imaging data during assessment of a suspected stroke patient. Such models can be useful in stroke management, especially in low-resource settings and can enable faster referrals of potentially eligible patients to higher stroke care centers. Further research with multicenter data and external validation is required to provide further corroborative evidence in this regard.

## Supporting information

S1 Table Acute infarct detection performance by radiologists and qER in noncontrast head CT scans. (DOCX)

S1 Fig MODEL_1_ predictors and their mean SHAP values. SHAP: SHapley Additive exPlanations.
(TIFF)

S2 Fig MODEL_2_ predictors and their mean SHAP values. SHAP: SHapley Additive exPlanations.
(TIFF)

S3 Fig Noncontrast CT head acquired from a suspected acute ischemic stroke patient for whom a mechanical thrombectomy was done at the site. CT perfusion (not shown in the figure) indicated 70 ml of tissue with cerebral blood flow (CBF) less than 30% and 133 ml of tissue with time to maximum (Tmax) of more than 6 seconds suggesting substantial amount (mismatch ratio: 1.9) of potentially salvageable tissue over the left middle cerebral artery territory. In the retrospective investigation, the acute infarct was detected by qER (drawn in red contours on the figure), but not by any of the four radiologists in the noncontrast CT scan.
(TIFF)
