# Supplementary material for: Development and internal validation of multimodal machine learning models for predicting eligibility for mechanical thrombectomy in suspected stroke patients using routinely collected clinical and imaging data
Source: PLoS One. 2025 Oct 10;20(10):e0334242. doi: 10.1371/journal.pone.0334242 (PMC12513648; doi:10.1371/journal.pone.0334242)
Supplement: S1 Table — (DOCX) [file pone.0334242.s001.docx]

**S1 Table.** Acute infarct detection performance by radiologists and qER in noncontrast head CT scans

| **Group** | **N^#^** | **Radiologist 1 (Neurorad)** | **Radiologist 2 (General rad)** | **Radiologist 3**  **(General rad)** | **Radiologist 4 (General rad)** | **qER** |
| --- | --- | --- | --- | --- | --- | --- |
| LVO_a_ and MT eligible | 160 | 13  (8.1 [4.4-13.5]) | 34  (21.2 [15.2-28.4]) | 10  (6.2 [3.0-11.2]) | 8  (5.0 [2.2-9.6]) | 40  (25.0 [18.5-32.4] |
| LVO_a_ but MT ineligible | 30 | 7  (23.3 [9.9-42.3]) | 10  (33.3 [17.3-52.8]) | 4  (13.3 [3.7-30.7]) | 6  (20.0 [7.7-38.6] | 18  (60.0 [40.6-77.3] |
| No LVO_a_ | 70 | 4  (94.3 [86.0-98.4] | 15  (78.6 [67.1-87.5]) | 5  92.8 [84.1-97.6] | 1  (98.6 [92.3-99.9] | 10  (85.7 [75.3-92.9] |

LVO_a_: anterior vessel large vessel occlusion; MT: Mechanical thrombectomy.

^#^ N indicates the number of NCCT scans (patients) in the group

Radiologist 1 was a neuroradiologist and all other three radiologists were general radiologists.

The numbers in the columns starting from ‘Radiologist 1’ indicate the number of acute infarct cases detected by the reader (N without parenthesis) and numbers in the parenthesis indicates – sensitivity in percentage and 95% confidence exact binomial confidence interval for the first two groups and specificity in percentage and 95% exact binomial confidence interval for the third group (No LVO_a_ and MT ineligible)
