# Supplementary figures and images for: Development and internal validation of multimodal machine learning models for predicting eligibility for mechanical thrombectomy in suspected stroke patients using routinely collected clinical and imaging data
Source: PLoS One. 2025 Oct 10;20(10):e0334242. doi: 10.1371/journal.pone.0334242 (PMC12513648; doi:10.1371/journal.pone.0334242)

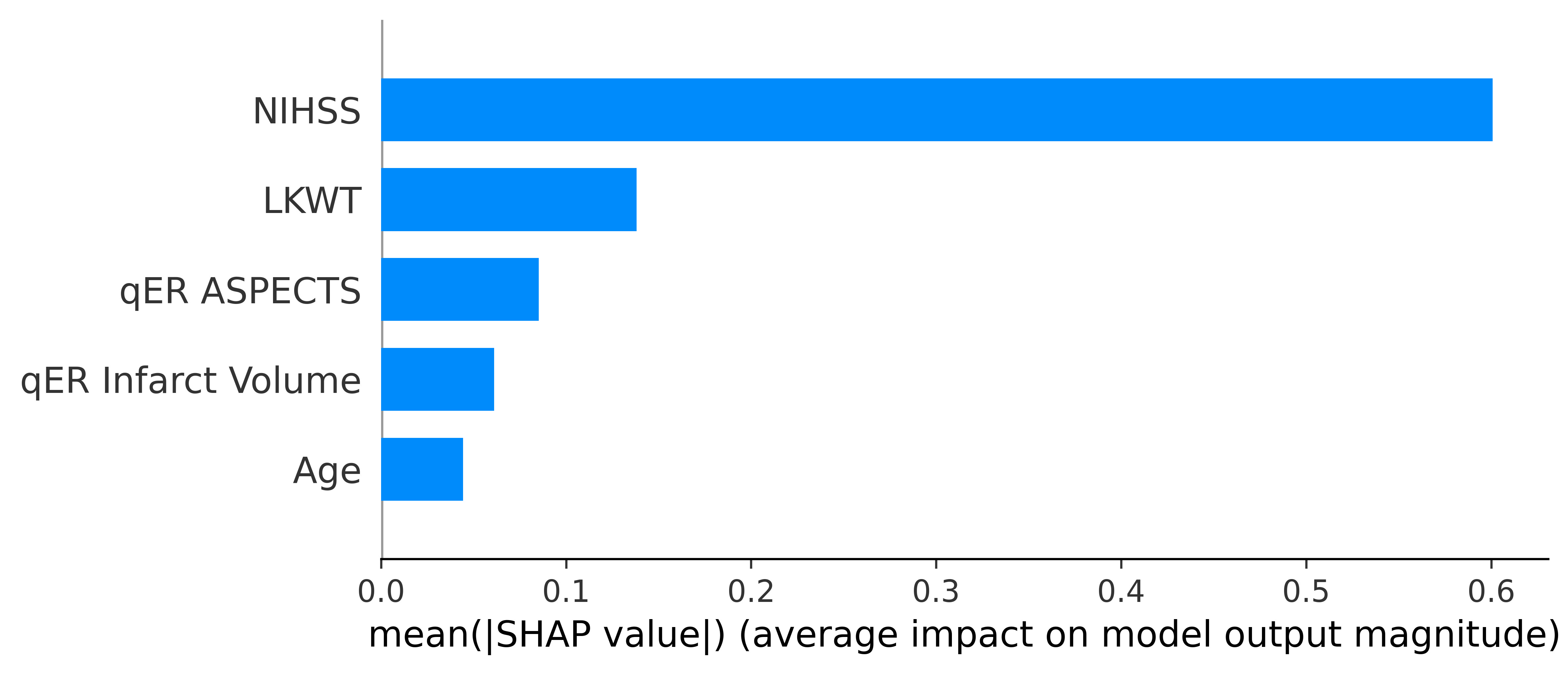

Supplement: S1 Fig — SHAP: SHapley Additive exPlanations. (TIFF) [file pone.0334242.s002.tiff]

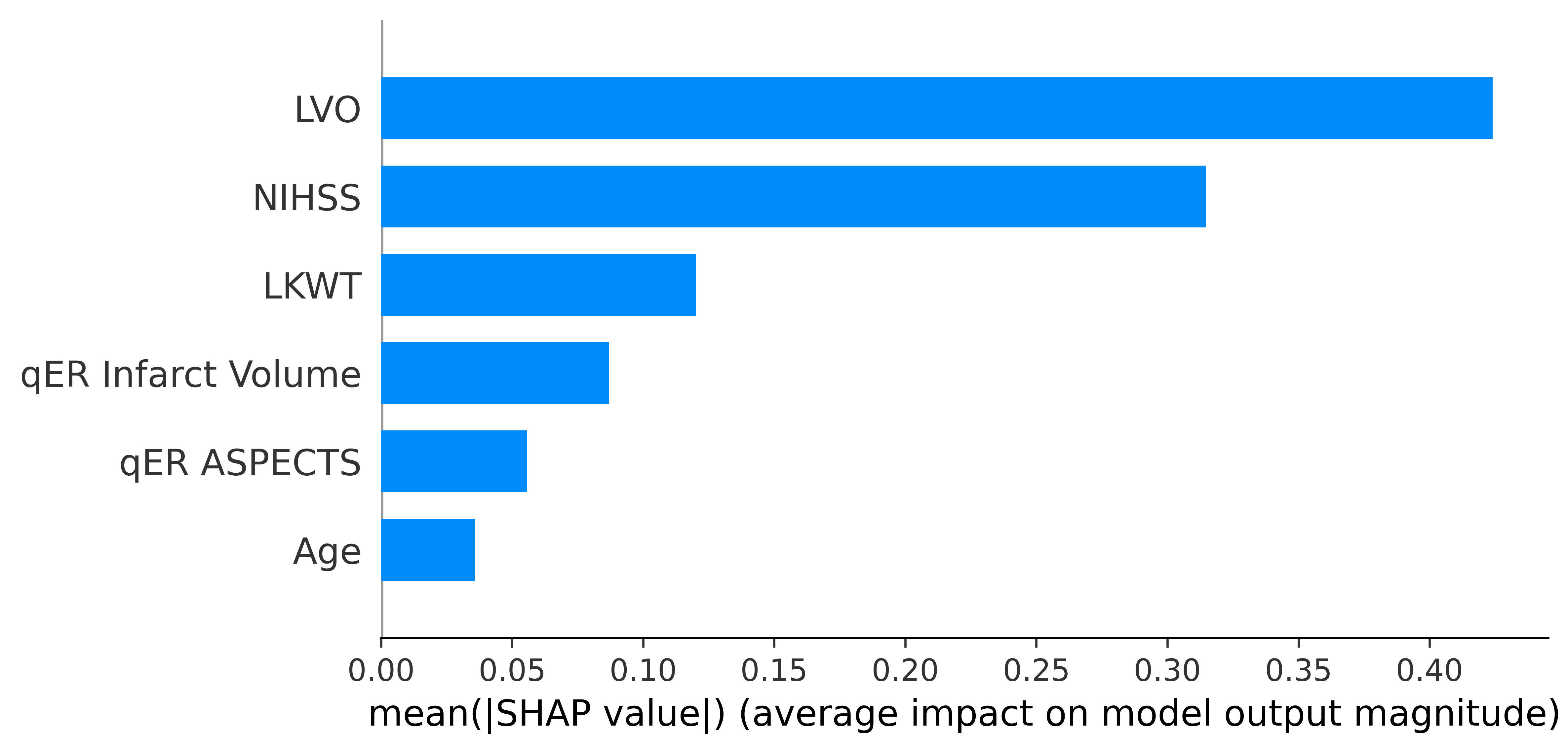

Supplement: S2 Fig — SHAP: SHapley Additive exPlanations. (TIFF) [file pone.0334242.s003.tiff]

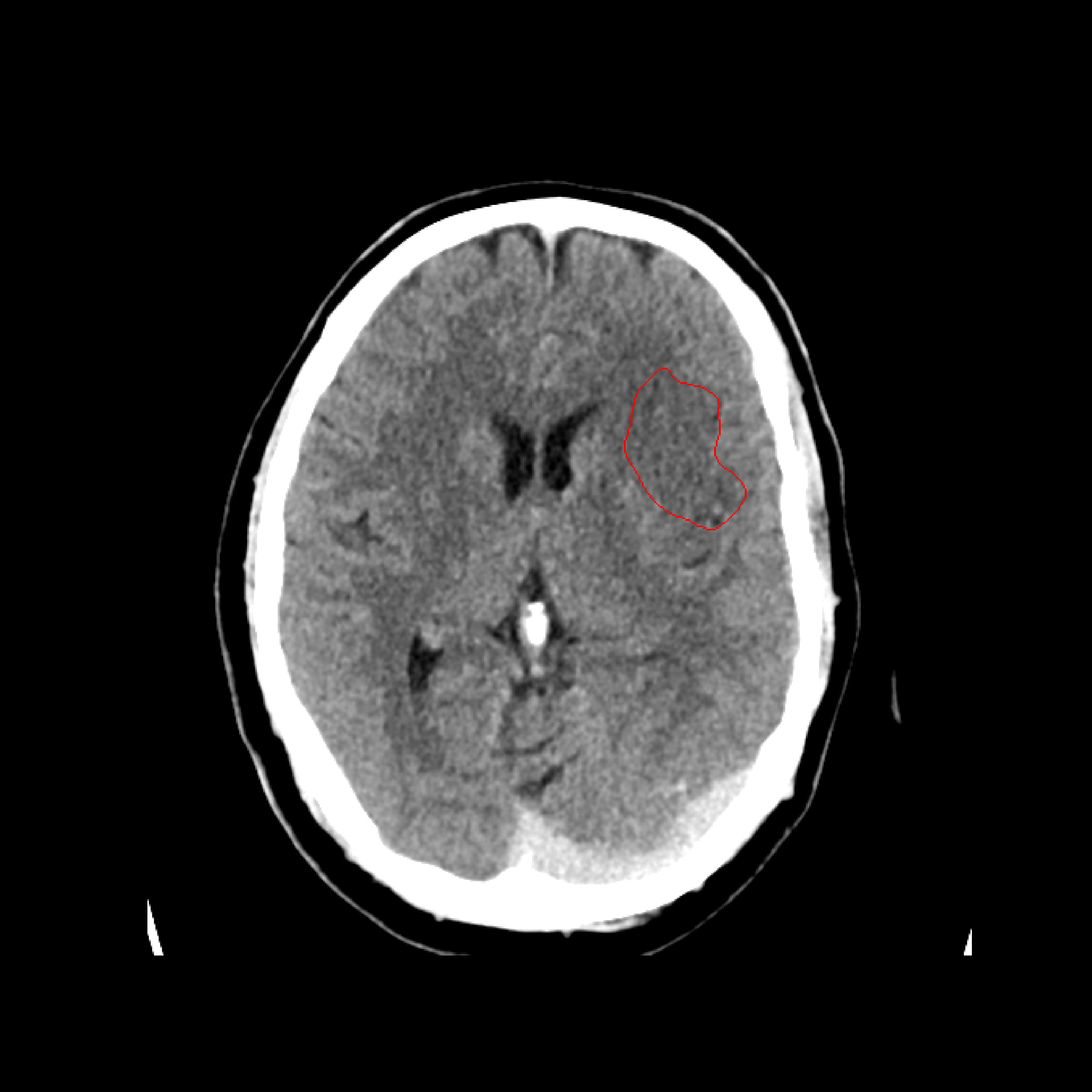

Supplement: S3 Fig — CT perfusion (not shown in the figure) indicated 70 ml of tissue with cerebral blood flow (CBF) less than 30% and 133 ml of tissue with time to maximum (Tmax) of more than 6 seconds suggesting substantial amount (mismatch ratio: 1.9) of potentially salvageable tissue over the left middle cerebral artery territory. In the retrospective investigation, the acute infarct was detected by qER (drawn in red contours on the figure), but not by any of the four radiologists in the noncontrast CT scan. (TIFF) [file pone.0334242.s004.tiff]
